# Therapeutic Notes

**Published:** 1896-05-30

**Authors:** 


### THERAPEUTIC NOTES.

### DUBOISIN AND SCOPOLAMIN.

The alkaloid duboisin has often been employed with advantage in cases of sleeplessness among the insane. The drug appears to be most efficacious when the insomnia is associated with intense motor excitement, and less effective or useless when the sleeplessness is accompanied by illusions, hallucinations, or organic brain mischief combined with motor disturbance. The doses which have been found sufficient for the promotion of sleep amount to about 1-80th of a grain. Scopolamin has more recently been tried as a substitute for the above, and in its effects it has been found almost identical. In sleeplessness[1] of insanity and in epilepsy it is reported to be of considerable value when administered hypodermically in doses of 1-50th to 1-30th of a grain.

The experiences of MM. Olderoggue and Jurmann[2] with regard to the value of the drug go to show that in chronic and acute trouble of psychic origin it has undoubted merits as a cerebral carminative and soporific. In acute cases the injection of the hydrobromate was, as a rule, followed by a refreshing sleep, before which event the pupil generally showed dilatation and the pulse less frequency. On waking the patient appeared still under the influence of the drug. Although the influence of scopolamin may be regarded as most satisfactory in cases of acute mania, its action is far less noticeable in melancholia associated with precordial distress. In delirium tremens it has no effect at all. In chronic condition the influence of the drug shows itself by quieting the cerebral excitement and inducing sleep. When the administration is continued too long unpleasant symptoms often manifest themselves, and for the most part these take the form of weakness in the legs, persistent mydriasis, occasional photophobia, slowing of the heart's action, and increased diuresis. Sometimes there is a feeling of weight in the head. Repeated injections of scopolamin are followed by a tolerance of the drug which makes it necessary to increase the dose, a practice which may be followed by one or more of the above symptoms, and hence the drug is obviously used to better purpose in acute cases.

[1] Ohmelevski, Neurol. Cent., Jan. 15, 1895. [2] Gazett de Hopitaux, March 21, 1896.

